# Characteristics of Cataracts in the Chinese Singaporean

**DOI:** 10.2188/jea.11.16

**Published:** 2007-11-30

**Authors:** Hiroshi Sasaki, Ying Bo Shui, Masami Kojima, Sek Jin Chew, Masaji Ono, Nobuyo Katoh, Hong-Ming Cheng, Nobuo Takahashi, Kazuyuki Sasaki

**Affiliations:** 1 Dept. of Ophthalmology, Kanazawa Medical University.; 2 Singapore Eye Research Institute, Singapore.; 3 Environmental Health Science Division, National Institute for Environmental Studies.; 4 Dept. of Hygiene and Public Health, Tokyo Women’s College.; 5 Retina Associates Foundation, Boston, Massachusetts, USA.

**Keywords:** epidemiology, UV-B, cataract, prevalence, nuclear cataract, scheimpflug photography

## Abstract

**Purpose:**

To conduct an epidemiological survey of cataracts and examine the characteristics of lens opacities in Chinese Singaporeans. The results are then compared with those from two similar surveys previously done in Japan in Noto Area, Honshu, and Amami, Kyushu, respectively.

**Subjects and Methods:**

468 subjects of ≥50 years of age, who responded to the invitation to participate, were examined. Examination principally included photo-documentation of the anterior and posterior segments of both eyes. Evaluation and grading of lens opacities were done using graphical analysis of Scheimpflug and retro-illumination images. Inter-group comparisons were based on statistical analysis of cataract prevalence and distribution.

**Results:**

The prevalence of clear lenses decreased with aging with no significant difference between males and females - a finding common to Singapore and the two Japanese study groups. The prevalence of cataracts (or lens opacities of Grade II and above) in 60-79 year-old Singapore subjects was significantly higher than Noto and Amami subjects in the same age group. Further, cortical opacity was the main type in Singapore subjects in their 50s and which was significantly higher than Noto subjects of the same age group. In all age groups, the distribution and prevalence of both nuclear and subcapsular types in the Singapore group were higher than the two Japanese study groups.

**Conclusions:**

Cataracts in Chinese Singaporeans are characterized by a high prevalence of nuclear opacities which was generally seen in tropics and sub-tropics. Our study also suggested the involvement of solar-UV in cortical cataracts as well as that of additional risk factors, such as environmental temperature and race, in nuclear and subcapsular cataract formation.

## INTRODUCTION

There appears an increase in age-related cataracts in recent years, not only in medically advanced nations but also in developing countries. It is an issue that needs to be addressed urgently; in fact, globally, cataracts as a cause of blindness have already afflicted 16 million people. The WHO Global Initiative calls for elimination of this cause of avoidable blindness by Year 2020^1^^)^. Its strategy is formulated specifically for developing countries. In contrast, cataract eradication plan for developed nations is still lacking; epidemiological surveys therefore must be conducted in order to gain a firm grip on the status of cataract prevalence and the identity of risk factors. Indeed, large-scale cataract surveys in developed nations have been or are being done^2^^-^^6^^)^. We have also conducted similar surveys, both inside Japan and on location outside of Japan, since the middle of 1980s^7^^-^^10^^)^. The causative factors of age-related cataracts and the compounding risk factors have been widely investigated. One of the recognized factors is solar ultraviolet-B (UV-B) exposure. It has been pointed out that due to the loss of the ozone layer in the Earth’s atmosphere, there has been an increasing UV-B irradiation and with which, an increase in the prevalence of UV-related eye diseases and skin cancer^11^^,^^12^^)^. We have recently continued both Japanese^9^^,^^10^^)^ and international surveys in part to study the effect of UV. Our domestic field study in Amami, Kagoshima-ken, Kyushu, in the south clearly showed that there were differences in lens pathology from that of the Noto Area, Honshu, in the central part of Japan. We now have an opportunity to conduct a comparative study in Singapore, where the ambient UV level was one of the highest in the world. The present report is in fact one link of an overall multi-site project. As a point of reference, the UV radiation in Singapore is 2 times higher than our home base, Noto Area in Ishikawa-ken. A broad-based comparative study should therefore yield useful information. For valid comparison, we used the same methodology as that for the previous studies. The first aim of our present Singapore study is to collect basic information on cataractous subjects and the second aim is to compare the results with that from Japan to identify common cataract patterns and possible risk factors, for example, if the uniquely high nuclear opacity prevalence found in the subtropical Amami is also present in the tropical Singapore and if the UV-exposure and other factors contribute to cataract prevalence.

## SUBJECTS AND METHODS

Climatic environment of the survey area: Singapore is an island located at the southern tip of Malay Peninsula, at latitude 1° 22′N and longitude 103° 55′E, with an altitude of 32m, an annual average temperature of 26.7°C and relative humidity of 86%, a rainfall of 2171.5mm/year, and UV-B radiation of 380 units/m^2^. For comparison, Noto and Amami in Japan are located, respectively, at latitude 36° 11′N and 28° 19′N, longitude 135° 23′ E and 130° 00′E, with an altitude of 5.6 and 2.8m above sea level, an average temperature of 15.1 and 21.3°C, average humidity of 71 and 74%, a rainfall of 2881 and 2870 mm/year, and a solar radiation of 190 and 240 units/m^2^, respectively.

Subjects: ≥50 year-old long-term Singapore residents of Chinese descent were surveyed. The invitation to participate was sent through Singapore Action Group of Elders (SAGE), which has an island-wide communication network. 517 subjects responded to 550 invitations - to designated dates of examination - and of which, 468 cases (male: 206 and female 262) were used for the analysis of lens change (Table 1). The number of the subjects (>50 year-old) for lens changes in Noto and Amami was 884 and 301, respectively.

**Table 1.** Patient composition and the number of eyes examined.

	Examined subjects			Analyzable cases of subjects			Analyzable eyes of subjects		

	Male	Female	Total	Male	Female	Total	Male	Female	Total
50 – 59	83	129	212	79	126	205	158	251	409
(years old)									
60 – 69	106	116	222	95	111	206	189	222	411
70 – 79	42	31	73	30	22	52	60	44	104
80 –	5	5	10	2	3	5	4	6	10

Total	236	281	517	206	262	468	411	523	934

Methodology: The same methodology as that used for surveys in Amami and Noto Area^9^^,^^10^^)^, and one other now ongoing in Reykjavik, Iceland, was employed.

Survey procedures: Prior to examination, a 26-item questionnaire (in both English and Chinese)^13^^)^ was distributed to and completed by the participants. Unfinished questionnaires were finished onsite with the assistance of survey staff. The participants were then subjected to the following: auto-refraction to determine refractive error, subjective distant visual acuity measurement, non-contact intraocular pressure test (Nidek NT-2000), undilated slit-lamp biomicroscopy of the anterior segment, Scheimpflug photography and graphic analysis system to ascertain the depth of the anterior chamber and the angle width, and specular microscopy (Konan) of corneal endothelium. Cases deemed suitable for pupillary dilation were instilled with topical Tropicamide 0.5% and Phenylephrine-HCl 0.5%. The lens and the fundus were examined under maximal dilation with slit-lamp biomicroscopy and the lens appearance was again recorded as Scheimpflug and retroillumination images with an anterior eye segment analysis system (Nidek EAS-1000). Fundus and the optic disc were recorded with conventional fundus camera (Topcon TRCNW5SF) and stereophotography (Nidek 3Dx/NM).

**Figure 1.**
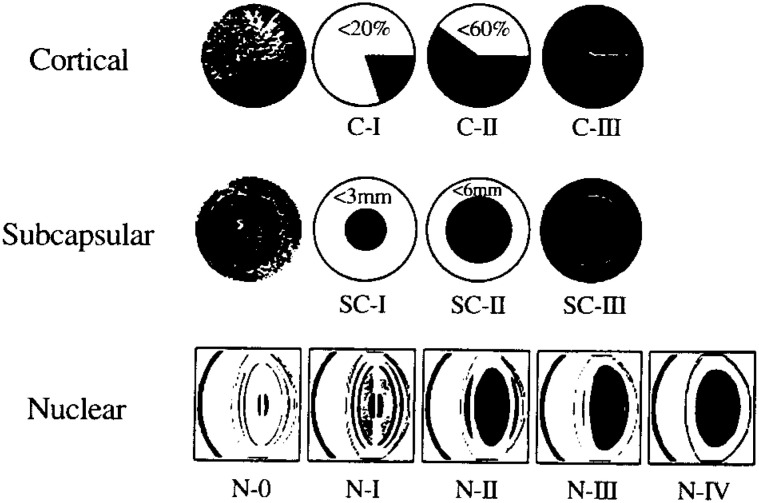
Classification and grading of lens opacities.

Classification and grading of lens opacities: These were based in principle on objective graphic analysis. The classification and grading of cortical and subcapsular opacities used Japanese Cataract Epidemiology Study Group System (JCCESG System)^14^^)^, nuclear opacities were classified according to Kanazawa Medical University Grading System from Grade I to IV^15^^)^. Previous reports used the same system except that Grades III and IV from which were now grouped together under Grade III (Fig. 1). Grades I, II and III of cortical opacities in the JCCESG System corresponded roughly with Lens opacities classification system II (LOCS II) Grades I, II - III and IV - V^16^^)^, respectively. Nuclear opacities in KMU System also corresponded roughly with LOCS I - IV. And subcapsular opacities Grade I, II and III in the JCCESG System corresponded with LOCS Grades I - II, III and IV respectively. Grade I opacities including very early change of lens opacity and usually does not impair the vision, while Grade II and III opacities often cause visual disturbance. Data collection followed the same methodology of previous studies^9^^,^^10^^)^. The final diagnosis of all cases were made by the same investigator who was well-versed in the classification system.

Study items: The present study concentrated on the prevalence and classification of lens opacities. Other aspects included the examination of risk factors, localization of cortical opacities, morphologic analysis of the exterior of the eye, observation of comeal endothelium, and detection of glaucoma. The results will be reported elsewhere. Prevalence analysis used only one eye from each patient that had pure cortical, nuclear or subcapsular opacities, or the mixed type. In cases where the two eyes differed, the more involved eye was used for analysis. If similar involvement but with different opacity types, the right eye was used. When comparing the prevalence of the three main types of lens opacities, i.e., cortical, nuclear and subcapsular, if both eyes had the same type of opacities but different grades, classification was done only for the eye with the higher opacity grade. Comparison of cataract types with previous surveys emphasized the distribution of cortical, nuclear, and subcapsular opacities using classification methodology mentioned above.

Statistics: Statistical analyses for the present study included t-test, *χ*^2^-test, and Mantel-Haenszel test. There was only 5 cases of >80 years old, too few to allow complete analysis. The data were used for reference only.

## RESULTS

### Cases available for analysis:

In the 517 participants, aphakes and pseudophakics (45 cases, 8.7%) and non-mydraitic cases ( 4 cases, 0.7%) were excluded for the analysis of lens changes. In two cases, analytical data between the two eyes were missing, two eyes only were used. The total usable cases were 468 and total eyes, 934 (Table 1).

### Prevalence of clear lenses:

**Figure 2.**
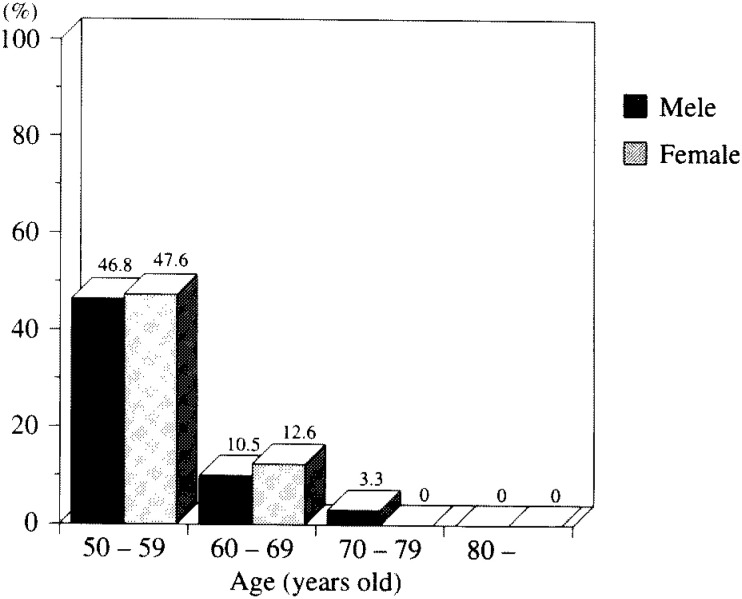
Prevalence of clear lenses.

Of the 468 cases, 122 (26.1%) were with both lenses clear. Subjects of over 60 years of age showed remarkably low prevalence compared with those in their 50s. There was, however, no significant difference between the two genders in all age groups (Fig. 2).

### Prevalence of lens opacities:

1) Prevalence of lens opacities of all types by gender and age

Figs. 3-a and 3-b show the prevalence of lens opacities by age in both males and females. The prevalence increased with increasing age in subjects with Grades I - III opacities (Fig. 3-a) and in those with above Grade I opacities (Fig. 3-b). For subjects over 60 years old, more than 50% showed apparent cataracts (i.e., opacities Grades II - III). On the other hand, no significant gender difference was found.

**Figure 3-a.**
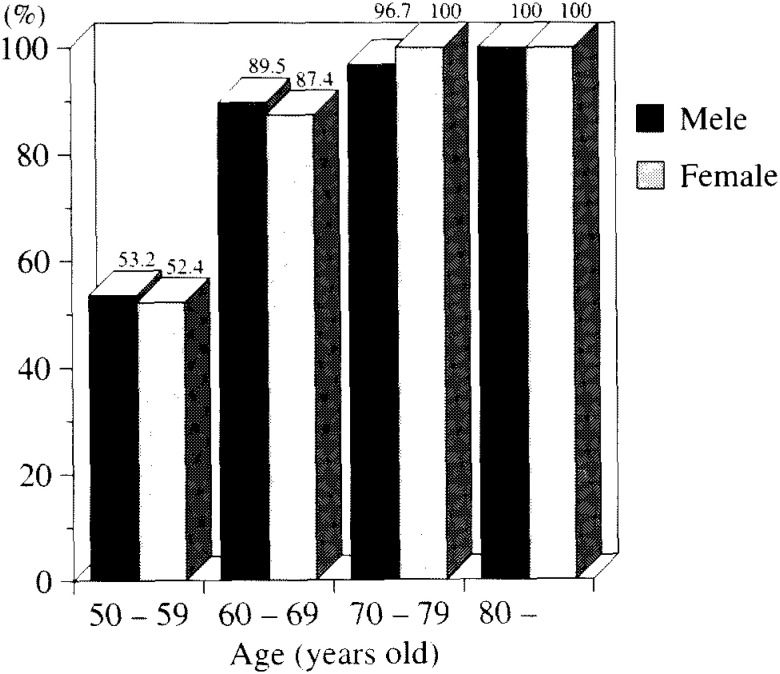
The proportion of lens opacities of all types from Grades I- III in the Singapore study group.

**Figure 3-b.**
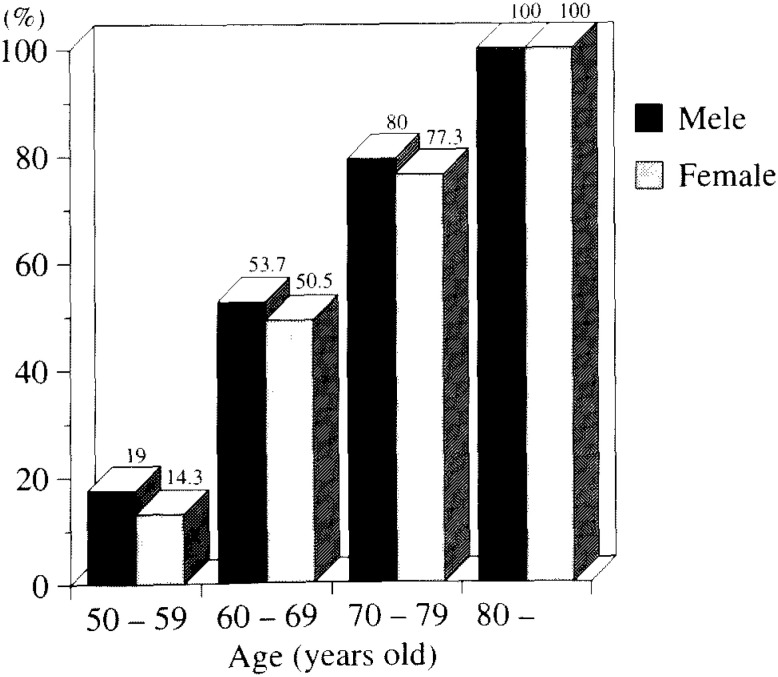
The proportion of lens opacities of all types from Grades II- III in the Singapore study group.

2) Prevalence of four different types of lens opacities by age and extent

The results are shown in Table 2. Prevalence of pure cortical opacity in subjects in their 50s was the highest among the four opacity types and it decreased with aging. In subjects over 60 years old, the prevalence of pure nuclear opacity was the highest among the three pure opacity types. Prevalence of the mixed type increased with aging reaching 40% in subjects over 60 years old. Prevalence of pure subcapsular opacities was extremely low in all age groups. The majority of subcapsular opacities was a component of the mixed type.

**Table 2.** The prevalence of the four lens opacity types by age.

		Type of cataract (%)			

Age (casea)	Grade	Pure Cortical	Pure Nuclear	Pure Subcapsular	Mixed
50 – 59	I - III	29.3	7.3	2.0	14.1
(205)	II - III	8.3	1.0	0.0	6.8
60 – 69	I - III	19.9	24.3	0.5	43.7
(206)	II - III	10.2	10.2	0.0	31.6
70 – 79	I - III	3.8	13.5	1.9	78.8
(52)	II - III	3.8	7.7	0.0	67.3
80 –	I - III	0.0	20.0	0.0	80.0
(5)	II - III	0.0	20.0	0.0	80.0

Average	I - III	22.0	15.6	1.3	35.0
(468 cases)	II - III	8.5	6.0	0.0	25.0

3) Distribution of three main types of lens opacities by age and gender

There was no significant difference in the distribution between males and females except for the cortical type in the age 60s group (Table 3). Cortical type was seen in 75 - 80% of subjects with lens opacities in all age groups; although distribution of the other two types did increase with aging.

**Table 3.** The distribution of the three main opacity types by age and sex.

(%)								

	Cortical				Nuclear		Subcapsular	

	Male		Female		Male	Female	Male	Female

50 – 59	76.2		87.9		45.2	39.4	16.7	9.1
(years old)								
60 – 69	68.6	*	81.4	*	80.2	73.2	17.4	14.4
70 – 79	89.7		68.2		93.1	90.9	55.2	31.8


* : P<0.05

4) Comparison with Noto and Amami groups

i) Retention rate of clear lenses

**Figure 4.**
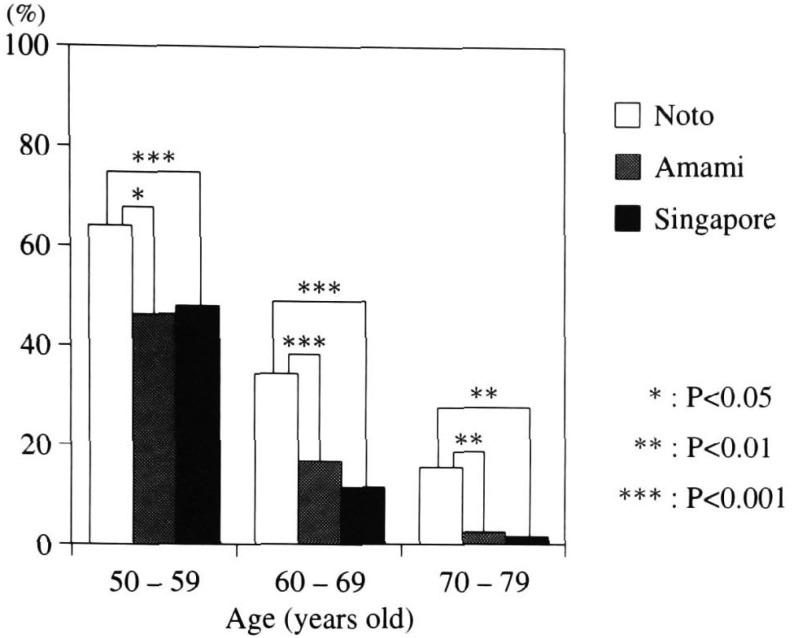
The prevalence of clear lens in all subjects in Noto. Amami and Singapore.

Comparison of the prevalence of clear lenses in the Singapore group with Noto^9^^)^ and Amami^10^^)^ groups is shown in Fig. 4. All age groups in Noto retained more clear lenses. The difference between Singapore and Amami was, however, insignificant.

ii) Prevalence of Grade II - III lens opacities of all types

**Figure 5.**
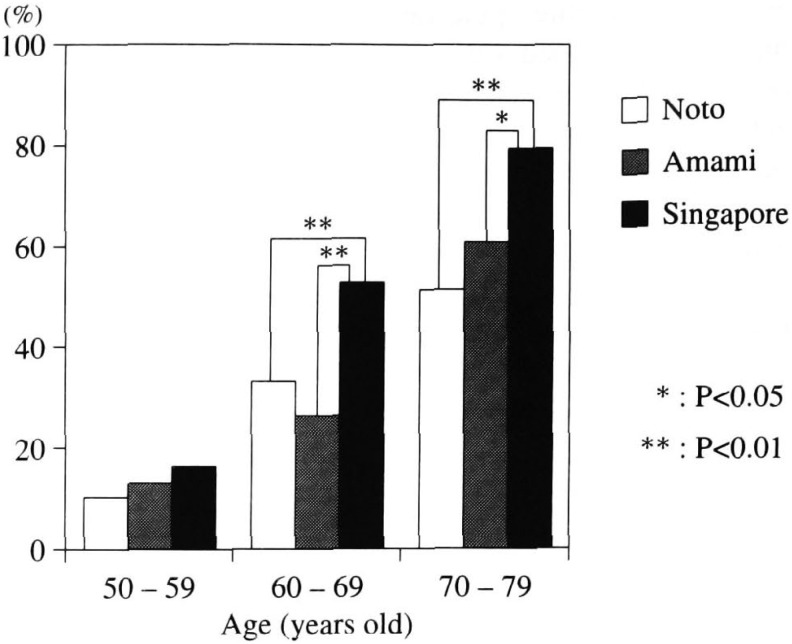
Comparison of prevalence of lens opacity of all types from Grades II-III among Noto, Amami and Singapore study groups.

Prevalence of Grade II and above opacities in the Singapore subjects was clearly higher than in Noto and Amami groups in both the 60s and the 70s age groups. (Fig.5).

iii) Distribution of lens opacity types

**Figure 6.**
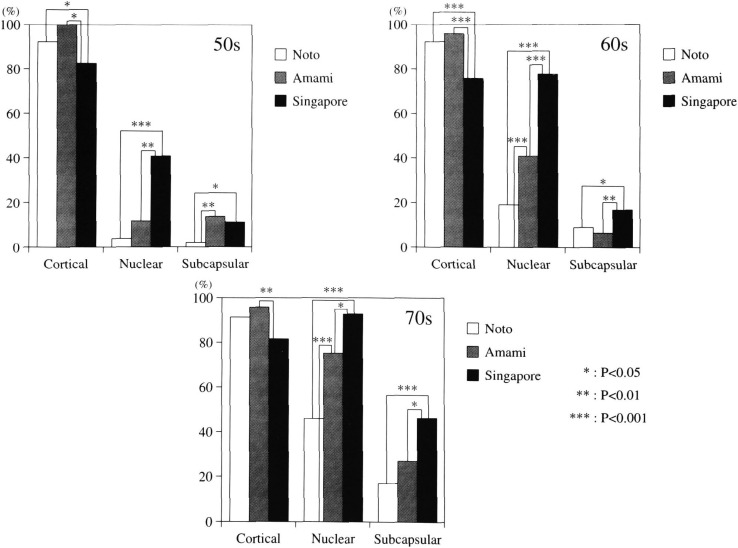
Distribution of three main opacity types in the subjects with lens opacity in three study groups.

**Figure 7.**
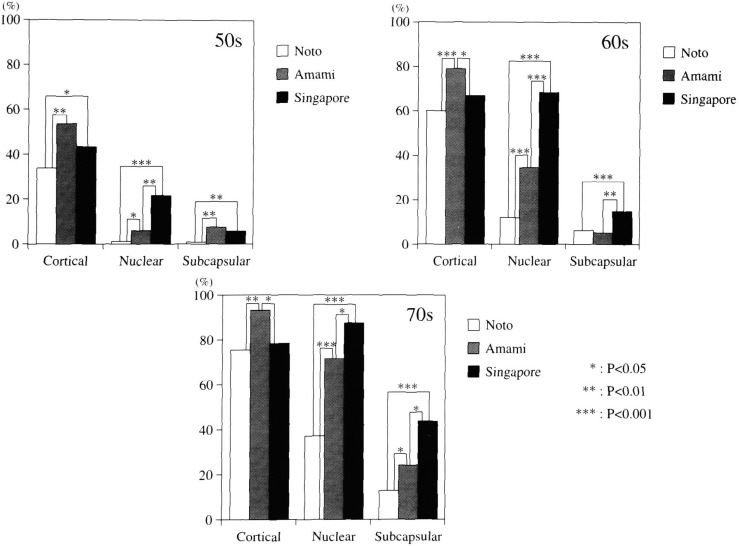
Comparison of the prevalence of three main types of opacities in all the subjects in three study groups.

The distribution of Grades I-III lens opacities is shown in Fig. 6. In subjects in their 50s in all three study groups, more than 80% of eyes with lens opacities had the cortical type; although Singapore subjects had the lowest distribution. Nuclear type was the highest in the Singapore subjects of all age groups. In the age 50s group, the distribution in the Singapore subjects was about 9 times higher than Noto and 3.5 times higher than Amami. The same tendency was seen in other age groups. Subcapsular opacity in Singapore subjects was significantly higher than Noto and Amami although the same between Amami and Singapore subjects all in their 50s.

iv) Prevalence of each opacity type with Grades I - III in all subjects (Fig. 7)

In subjects in their 50s, the prevalence of cortical opacity in Noto subjects was lower than the other two study groups. For subjects over 60 years of age, the prevalence in Amami was significantly higher than the other two study groups. In all age groups, the prevalence of nuclear opacity in the Singapore group was significantly higher than the two Japanese study groups; in fact, the Singapore group was 13 times higher than Noto in subjects in their 50s, 6 times higher in the age 60s group, and 2.5 times higher in the 70s or higher age groups. The prevalence of subcapsular type increased with aging; in all age groups, the Singapore study group was significantly higher than the Noto group and in the ages 60s and 70s groups, significantly higher than those in Amami.

## DISCUSSION

The present study was population-based although not an ideal randomized study. The purpose of the study was conveyed to the public initially through civic organizations and later reinforced by newspaper and TV news reports; although a pre-determined number of Chinese only was admitted to the study. There was, however, no bias in geographic selection and patient choice. The present study was also limited to the Chinese who constitute 77% of the Singaporeans. The prevalence of cataracts is not expected to be grossly affected by minor variations in the living habits of the same ethnic group.

Age-related cataract is a result of lens physiological change compounded by multiple risk factors. This age-related change also must take geographic and racial factors into consideration. The prevalence of clear lenses was similar to residents of the sub-tropical Amami. The basis of physiological aging change is therefore similar for both Singapore and Amami (Fig. 4).

When comparing the presence of lens opacities of Grade II and above in Singapore with that in Amami and the Noto Area, there was a significant difference in subjects in their 60s and 70s - the Singapore group clearly had a significantly higher prevalence than the other two study groups. When comparing the prevalence of cataract of all types with Grades II and III opacities, Singapore subjects in their 60s roughly corresponded with the Noto subjects in their 70s. These results suggest that the progression of cataract in the Chinese Singaporeans might be faster than that of the Noto residents. Although this finding may imply a solar-UV cataract promoting effect, there has been no definite conclusion as far as UV-specific lens opacity types. Indeed, the correlation with nuclear and subcapsular opacity types should be further probed. In comparing the prevalence and distribution of the three opacity types in cataracts of the three study groups, several interesting findings emerged: Overall, it is clear that the distribution of each opacity type in different study groups has its own characteristics. Cortical type in the age 50s group was seen in over 80% of opacified lenses in three climatically different locales. The prevalence of cortical type in Singapore subjects in their 50s was significantly higher than the Noto subjects (p<0.05). From the standpoint of UV causation of cortical cataracts, which has been experimentally proven, it is logical to postulate that the high dose of solar UV irradiation in Singapore has in fact influenced the appearance and/or progression of cortical cataracts. Since the appearance of lens opacities generally starts in the 5th decade of life, cortical cataract as a main opacity in this age group in the Singapore subjects is therefore expected. What was unexpected was that the main type shifted to nuclear type in subjects older than 60 years. Prevalence and distribution of nuclear type in the Singapore group was significantly higher than those in the Amami and Noto groups. The similarity of prevalence and distribution of nuclear cataract between Amami and Singapore may be related to yet to be identified risks factors those are common to both areas.

Increase of subcapsular opacity in the mixed type with aging is well recognized. Even though the prevalence of pure subcapsular opacity is very low, we have found that it is a major component of the mixed type. Distribution of subcapsular opacity was the highest in Singapore followed by Amami and that in Noto was extremely low. At present, the causative factors of this type of opacity are still unclear. The relationship between solar-UV and subcapsular cataract merits further studies.One of the study purposes was to ascertain the characteristic presence of nuclear cataracts in residents in the tropics and the subtropics, the Singapore group indeed has unusually high prevalence of this type of opacity. The reason for this occurrence is at present unclear. Excepting for solar UV, several other factors such as race, living habits and environmental temperatures may also be involved. We had examined these possibilities in a separate survey in Sumatra, Indonesia^8^^)^. We used the same graphical analysis of lens opacity types and grading and the results showed that the nuclear opacity prevalence had a tendency similar to that observed in Singapore and that race might remain a factor in determining lens opacity types. The common factors in living conditions of these two locations appear to be high UV exposure and high ambient temperature. There have been few studies on the appearance of cataracts and the effect of higher environmental temperature on the degree and the prevalence of nuclear opacities^17^^,^^18^^)^. The effect of high temperature on cataract formation also must be further investigated.

Interestingly, even though Singapore has high solar UV radiation, it seemed unrelated to the prevalence of cortical cataracts in subjects over 60 years old. Cortical cataract, however, is still the main opacity type in subjects in their 50s. According to our risk factor search case-control study, long duration of daily outdoor activities correlates significantly with the appearance of cortical cataracts^19^^)^. Preliminary results showed that the time spent outdoors was significantly longer for the two Japanese study groups than the Singapore group. We speculate that this might be one of the reasons why the unexpected low prevalence of cortical opacities in older Singapore subjects. Another possibility maybe that the outdoor time did not coincide with the duration of maximal UV exposure (i.e., total dose), hence the difference between the 50s group and older groups.

We should point out that, in terms of overall cataract progression in Chinese Singaporeans, we estimate it being 10 years sooner than Noto residents. This information should be useful in the planning for cataract management in Singapore. Further, the high prevalence of nuclear opacities in the tropics and the subtropics has now been confirmed. The contributing effect of UV to nuclear and subcapsular opacification, however, remains to be further investigated.
